# Photobiomodulation mitigates *Bothrops jararacussu* venom-induced damage in myoblast cells by enhancing myogenic factors and reducing cytokine production

**DOI:** 10.1371/journal.pntd.0012227

**Published:** 2024-05-30

**Authors:** Luciana Miato Gonçalves Silva, Viviane Almeida Gouveia, Gabriela Russo Soeiro Campos, Camila Squarzone Dale, Renata Kelly da Palma, Ana Paula Ligeiro de Oliveira, Rodrigo Labat Marcos, Cinthya Cosme Gutierrez Duran, José Carlos Cogo, José Antônio Silva Junior, Stella Regina Zamuner

**Affiliations:** 1 Postgraduate Program in Medicine, Universidade Nove de Julho, UNINOVE, São Paulo, Brazil; 2 Postgraduate Program in Medicine-Biophotonics, Universidade Nove de Julho, UNINOVE, São Paulo, Brazil; 3 Department of Anatomy, Institute of Biomedical Sciences, University of São Paulo, São Paulo, São Paulo, Brazil; 4 Facultad De Ciencias De la Salud de Manresa, Universitat de Vic-Universitat Central De Catalunya (UVic-UCC), Barcelona, Spain; 5 Tissue Repair and Regeneration Laboratory (TR2Lab), Institute for Research and Innovation in Life and Health Sciences in Central Catalonia (Iris-CC). Vic, Spain; 6 Programa de Mestrado em Bioengenharia do Instituto de Ciências e Tecnologia da Universidade Brasil, São Paulo, Brazil; Instituto Butantan, BRAZIL

## Abstract

**Background:**

Photobiomodulation has exhibited promise in mitigating the local effects induced by *Bothrops* snakebite envenoming; however, the mechanisms underlying this protection are not yet fully understood. Herein, the effectiveness of photobiomodulation effects on regenerative response of C2C12 myoblast cells following exposure to *Bothrops jararacussu* venom (BjsuV), as well as the mechanisms involved was investigated.

**Methodology/Principal findings:**

C2C12 myoblast cells were exposed to BjsuV (12.5 μg/mL) and irradiated once for 10 seconds with laser light of 660 nm (14.08 mW; 0.04 cm^2^; 352 mW/cm^2^) or 780 nm (17.6 mW; 0.04 cm^2^; 440 mW/ cm^2^) to provide energy densities of 3.52 and 4.4 J/cm^2^, and total energies of 0.1408 and 0.176 J, respectively. Cell migration was assessed through a wound-healing assay. The expression of MAPK p38-α, NF-Кβ, Myf5, Pax-7, MyoD, and myogenin proteins were assessed by western blotting analysis. In addition, interleukin IL1-β, IL-6, TNF-alfa and IL-10 levels were measured in the supernatant by ELISA. The PBM applied to C2C12 cells exposed to BjsuV promoted cell migration, increase the expression of myogenic factors (Pax7, MyF5, MyoD and myogenin), reduced the levels of proinflammatory cytokines, IL1-β, IL-6, TNF-alfa, and increased the levels of anti-inflammatory cytokine IL-10. In addition, PBM downregulates the expression of NF-kB, and had no effect on p38 MAKP.

**Conclusion/Significance:**

These data demonstrated that protection of the muscle cell by PBM seems to be related to the increase of myogenic factors as well as the modulation of inflammatory mediators. PBM therapy may offer a new therapeutic strategy to address the local effects of snakebite envenoming by promoting muscle regeneration and reducing the inflammatory process.

## Author summary

Photobiomodulation (PBM) therapy, a technique using light to influence cell behavior, has shown promising results in reducing the damage caused by snakebites from *Bothrops* species, a common venomous snake in Latin America. This study explores how PBM aids in the recovery of muscle cells after exposure to *Bothrops jararacussu* venom. Our research focused on C2C12 myoblast cells, a type of muscle cell, treated with PBM following venom exposure. We observed that PBM not only helped these cells move and close wounds faster but also increased the production of proteins essential for muscle formation and recovery. Interestingly, it also shifted the balance towards less inflammation by decreasing pro-inflammatory signals and increasing anti-inflammatory ones. This work sheds light on the potential of PBM as a novel treatment approach for snakebite victims. By promoting muscle repair and reducing inflammation, PBM therapy could improve recovery outcomes following envenoming. Our findings suggest that, with further development, PBM could become a valuable tool in the medical response to snakebites, offering a ray of hope for faster, more effective recovery processes.

## Introduction

It is well documented that snakebite envenoming is of clinical and social importance worldwide. Snake envenoming affects thousands of individuals every year mainly in tropical and subtropical areas of the world [1,2]. In addition to death, many snakebite patients develop permanent physical and psychological sequelae that greatly affect their quality of life [3]. The species that belong to the genus *Bothrop*s are among the snakes of greatest clinical interest in Latin America accounting for 50–90% of the snakebites [4]. Local injury on the skeletal muscle is a common manifestation due to fast-developing injury at the bite site, which can lead to necrosis, dysfunction and in some cases amputation [5]. The recommended treatment for envenomation is the administration of antivenom therapy, which may prevent death, but does not prevent local tissue damage and subsequent functional disabilities making it necessary to search for other therapeutic strategies [6].

Photobiomodulation therapy (PBMT) emerges as a promising noninvasive treatment for musculoskeletal disorders, including tendinopathies [7], fibromyalgia [8], and muscle injuries [9]. This approach involves the irradiation of biological tissues with low-level laser (LLL) or light-emitting diode (LED), contributes to tissue regeneration, accelerating healing, and pain relief. The stimulation of cellular processes, enhancement of biochemical mechanisms, and reduction of inflammation are key contributors to its therapeutic effects [10]. Furthermore, the literature suggests the potential of PBMT as an alternative therapy for mitigating local effects induced by *Bothrops* genus snakebites, demonstrating its capacity to attenuate venom-related damage [4]. Moreover, a recent randomized clinical trial demonstrated that PBMT is feasible and effective in reducing myonecrosis and local inflammatory effects, such as pain and edema, caused by *B*. *atrox* envenomations [11]. However, it is important to highlight that existing investigations have not yet provided a comprehensive assessment of how PBMT specifically influences muscle repair following venom exposure.

Muscle regeneration means the biological repair and formation of new muscle tissue in response to the necrosis of muscle cells [12]. The intricate process of skeletal muscle repair unfolds through a series of phases, initiating with degradation and inflammation, progressing to an extensive repair stage and concluding with maturation and remodeling [13]. PBMT has been reported to exert a beneficial influence on muscle regeneration. This is achieved through its capacity to stimulate the proliferation of muscle and satellite cells, enhancing protein synthesis in myoblasts, increasing the area of muscle fibers, enhance mitochondrial density [14–17], and upregulate the expression of myogenic regulatory factor, including pax7, myoD, and myogenin [15,18–19].

While the data supporting the efficacy of Photobiomodulation (PBM) therapy for Bothrops snakebites appears promising, there remains a gap in understanding the correlation between the myonecrosis induced by these venoms and the subsequent tissue repair facilitated by PBM. Therefore, we examine the ability of PBM to protect C2C12 cells from the effects of *Bothrops jararacussu* venom, while also exploring the potential mechanisms responsible for this protective effect.

## Material and methods

### *B*. *jararacussu* venom

*B*. *jararacussu* venom was supplied from the Center of Studies of the Nature at UNIVAP. The venom was lyophilized and kept under refrigeration at 4°C, being diluted in culture medium immediately before use.

### Cell line and culture conditions

The murine C2C12 cell line (ATCC) was used as the venom target. For maintenance of C2C12 myoblast, cells were cultured in growth medium consisting of Dulbeccos modified Eagles medium (DMEM, Cultilab, Campinas, SP, Brazil) supplemented with heat-inactivated 10% fetal bovine serum (FBS) and 1% antibiotic-antimycotic solution in a humidified atmosphere of 5% CO2 at 37°C. Growth medium was changed every two days.

### Experimental groups

Myoblast cell cultures were used in all experiments and all measurements were obtained from triplicate cultures. The following groups were studied: (1) Control (cells non-irradiated); (2) BjsuV (cells incubated with *B*. *jararacussu* venom); (3) BjsuV + 660 nm (cells incubated with venom and immediately irradiate with laser at 660 nm); (4) BjsuV + 780 nm (cells incubated with venom and immediately irradiate with laser at 780 nm). The venom dose used was 12.5 μg/mL and was chosen on the basis of previous study from our group which showed that a dose of 12.5 μg/mL decrease 50–60% cell viability in the period of 15 to 60 min [15,20].

### Laser irradiation

The experiments utilized semiconductor lasers, specifically gallium aluminum arsenide (Ga-AlAs) at 780 nm in the near-infrared range, and aluminum gallium indium phosphide (InGaAlP) at 660 nm, in red (MM Optics Ltd., São Carlos, SP, Brazil). The parameter settings are detailed in Table 1. Following the addition of venom to the cell culture, cells were immediately irradiated. The laser beam was positioned perpendicular to the lower surface plate, focusing on a single point at the center of each culture well without relocating the laser tip. The experiments were conducted in a partially obscured environment to avoid interference from external light. The optical power output of the laser was measured using a Newport multifunction optical meter (model 1835C, Newport Corp., Irvine, CA, USA).

**Table 1:** Laser parameter setting.

*Parameter*	*Red laser*	*Near-infrared laser*
Wavelength (nm)	660	780
Average radiant Power (mW)	16	20
Effective radiant Power (mW)	14.08	17.6
Operating mode	Continuous	Continuous
Beam spot size (cm^2^)	0.04	0.04
Area irradiated (cm^2^)	0.04	0.04
Irradiance (mW/ cm^2^)	400	500
Radiant exposure (J/cm^2^)	4	5
Effective radiant exposure (J/cm^2^)	3.52	4.4
Exposure duration (s)	10	10
Total energy (J)	0.16	0.2
Effective total energy (J)	0.1408	0.176
Number of points irradiated	1	1
Application technique	Contact	Contact
Number of treatment sessions	1	1

### Migration assay

Cell migration was assessed through a wound-healing assay. In brief, cells were cultured in a 6-well plate (2 x 10^3^ cell/mL) until reaching ~90% confluence at 37°C in a 5% CO2 incubator. Once confluence was verified, the scratch assay was carried out, creating the wound, straight line was made in the medial region of the plaque using a tip, providing a rupture between the cells, causing a mechanical injury. To remove debris resulting from the lesion, the culture medium was discarded and then the wells were washed twice with phosphate-buffered saline (PBS 1x), and new DMEM medium was added followed by incubation with or without *B*. *jararacussu* venom (12.5 μg/mL) and by immediate application of PBM (660- or 780 nm), and the cells were ncubated for 1 hour. After this incubation period, the cultured cells were washed to remove the venom, and the medium was replaced. The migration of myoblast cells into the wounded area was evaluated using photographs (50x) at 0 and 24 h later. The images were captured with a digital camera attached to a Zeiss inverted microscope. The number of cells that migrated to the wound was quantified using ImageJ software [21], and the percentage of cell migration was calculated by comparing it with the control group, considered as 100% migration. Each sample was assayed in triplicate wells, from three independent experiments.

### Western blotting

To assess the presence of p38 MAPK, NF-Кβ, Myf5, Pax-7, MyoD, and myogenin proteins in C2C12 cells following incubation with Bjssu (12.5 μg/mL) and laser irradiation, western blotting was employed. To assess the presence of p38 MAPK, NF-Кβ, Myf5, Pax-7, MyoD, and myogenin proteins in C2C12 cells following incubation with BjssuV (12.5 μg/mL) and laser irradiation, western blotting was employed. The evaluation was conducted after 6 hours. The selection of the 6-hour incubation period for assessing the expression of p38 MAPK was based on previous studies demonstrating a significant increase in various cytokine levels over this time frame, including IL-6 [14], known to activate p38 MAPK in muscle cells. After 6 h of venom incubation, cells were rinsed with cold phosphate-buffered saline (PBS) and lysed directly using Laemmli sample buffer. The resulting samples underwent centrifugation (10,000 x g for 15 min), and the protein concentration in the supernatant was determined through a colorimetric assay (BioRad, USA). Subsequently, 30 μg of protein was separated on a 10% SDS-PAGE gel and transferred to a nitrocellulose membrane (Bio-Rad–USA). The membrane was then blocked for 1 hour at room temperature using blocking buffer (20 mM Tris, 100 mM NaCl, 5% non-fat milk, and 0.5% Tween-20). Following blocking, the membrane was incubated overnight at 4°C with antibodies targeting MAPK p38-α (1:1000, Cell Signaling Technology-USA), NF-kb (1:1000, Cell Signaling Technology-USA), Myf5 (1:1000, Cell Signaling Technology-USA), Pax-7 (1:1000, Cell Signaling Technology-USA), MyoD (1:1000, Cell Signaling Technology-USA), Myogenin (1:2000, Cell Signaling Technology-USA), or β-actin (1:1000, Cell Signaling Technology-USA). Following an additional wash, the membrane underwent incubation with an appropriate horseradish-peroxidase-conjugated secondary antibody at room temperature for 2 hours. Immunoreactive bands were visualized using a chemiluminescence detection system (Scanner Storn 860 Molecular Imager). The relative band intensity was quantified using ImageJ software [21] after normalizing with β-actin band intensity. Each sample was assayed in triplicate wells, from three independent experiments.

### Quantification of inflammatory cytokines in the cell supernatant

Cytokine levels (IL-1β, IL-6, TNF-alpha, and IL-10) in the cell supernatant of C2C12 cells following BjsuV incubation and PBM irradiation were measured through enzyme-linked immunosorbent assay (ELISA), following the manufacturer’s guidelines (BioLegend, San Diego, CA, USA). The outcomes were quantified in picograms (pg) of the respective cytokine produced per milliliter (mL) of supernatant. Each sample was assayed in triplicate wells, from three independent experiments.

### Statistical analyses

To verify normality and error variances, the Shapiro–Wilk test was used. The results indicated a normal distribution for both experimental conditions (BjV p = 0.943, venom + PBM p = 0.926). Comparisons among the experimental groups were conducted using one-way ANOVA, followed by the Student’s Newman-Keuls test for multiple comparisons using the GraphPad prism software V.5. P values less than 0.05 were considered statistically significant, and results were presented as mean ± standard error of the mean (SEM).

## Results

### Effect of PBM on C2C12 Myoblast cell migration

**Fig 1:**
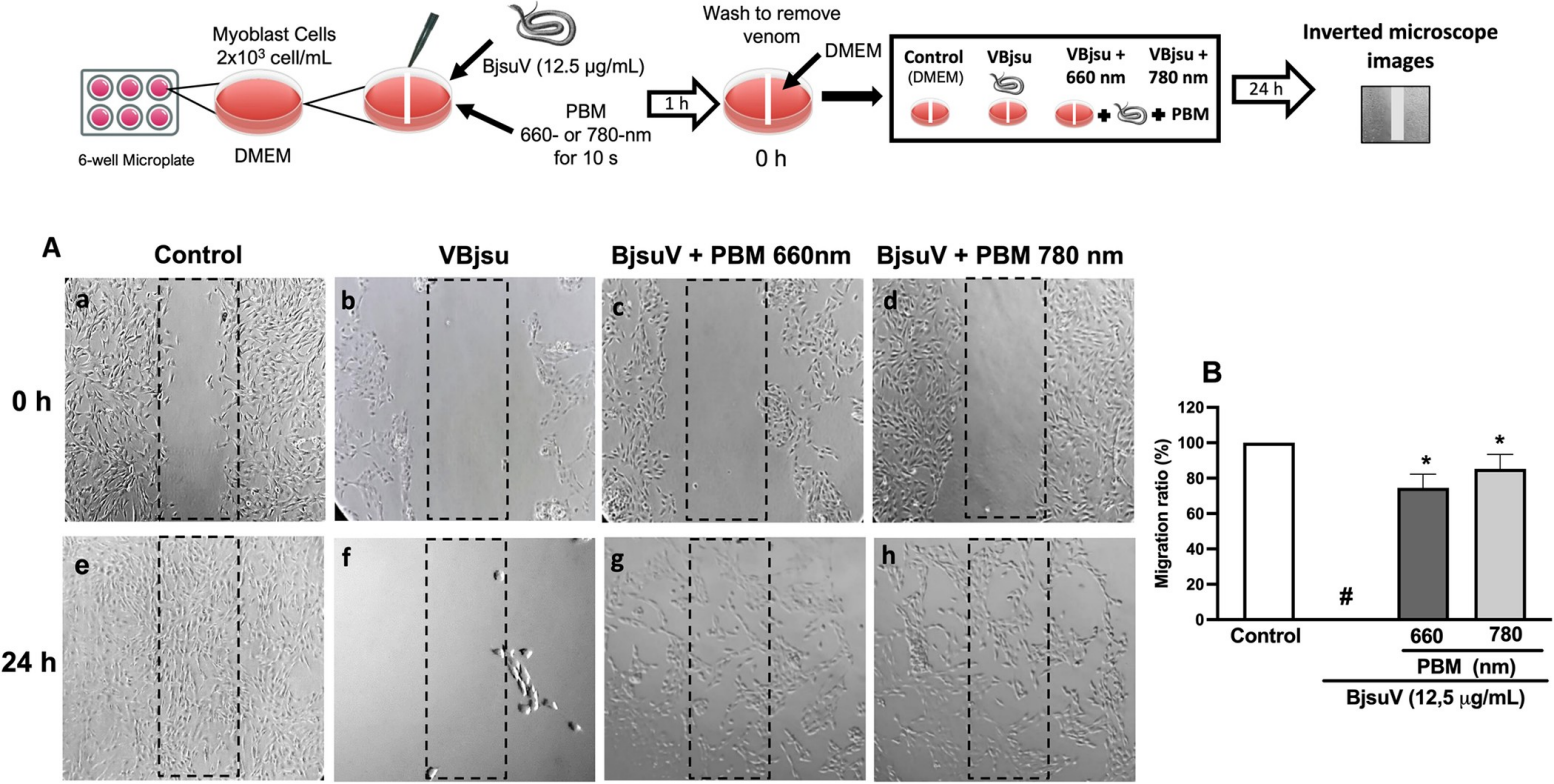
Effect of photobiomodulation on the migration of C2C12 myoblasts cells. Cells were seeded in plates and then incubated with 12.5 μg/mL of BjsuV in the presence or absence of PBM irradiation for 1 h. Then cells were washed, the medium replaced, scratched with micropipette and then incubated for 24 h. Then phase contrast microscope was used to take the photos (50x). The representative pictures are shown in A (a-h). Cells migration is represented as percentage of the cell that migrated to the scratched area. The scratched area was quantified by ImageJ software and data are shown in B. The picture represents one independent experiment (n = 3). #*P* < 0.05; compared to control; *P < 0.05; compared with the BjsuV group. BjsuV, *Bothrops jararacussu* venom; PBM, photobiomodulation. Figure diagram built using https://commons.wikimedia.org as source of the imagens or icons.

A wound-healing assay was conducted to assess the impact of PBM on the migratory behavior of C2C12 cells. Our observations revealed that the wound scratch in control cells exhibited complete closure after 24 hours of incubation (Fig 1Ae). Conversely, following 24 hours of venom incubation, no viable cells were evident (Fig 1Af). However, PBM irradiation demonstrated the capacity to promote cell migration within the wound area, achieving a 76% migration at 660 nm (Fig 1Ag and 1B) and an 83% migration at 780 nm (Fig 1Ah and 1B).

### Effect of PBM on the expression of myogenic regulatory factors

**Fig 2:**
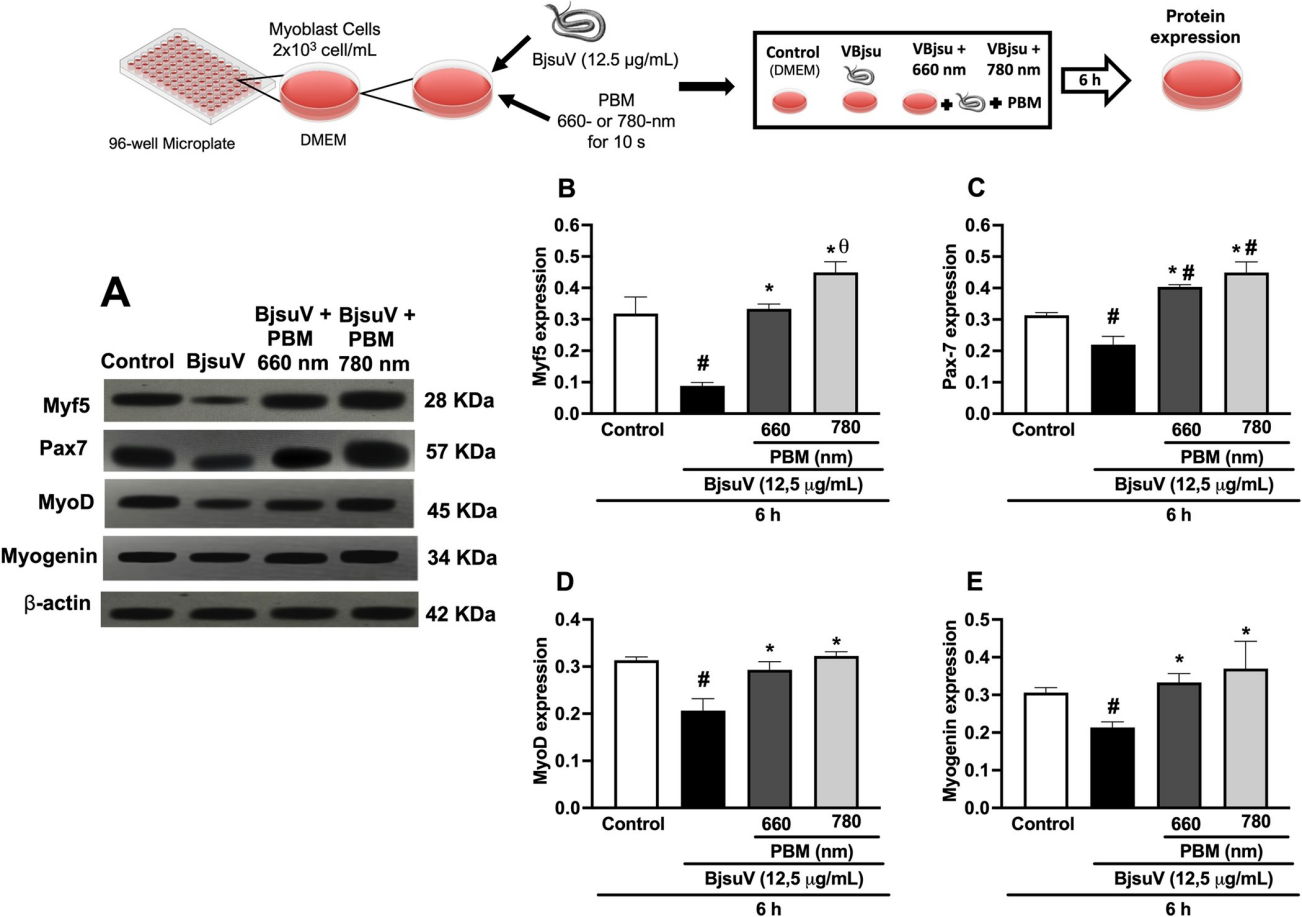
Effect of photobiomidulation on the expression of myogenic regulatory factors. Representative immunoblot analyses and corresponding bar graphs depicting the protein levels of Myf5 (A and B), Pax7 (A and C), MyoD (A and D), and Myogenin (A and E) in C2C12 myoblast cells. The cells were incubated with 12.5 μg/mL of BjsuV, and the impact of photobiomodulaton treatment was assessed after 6 hours. β-actin served as the loading control. All experiments were performed in triplicate, and the relative intensity of the bands was quantified. Each value represents the mean ± SEM of three independent experiments, #p< 0.05 vs the control group; *p< 0.05 vs the venom-exposed group. BjsuV, *Bothrops jararacussu* venom; PBM, photobiomodulation. Figure diagram built using https://commons.wikimedia.org as source of the imagens or icons.

The Western blot analysis presented in Fig 2A explores into the levels of key myogenic factors—Myf5, Pax7, MyoD, and myogenin—crucial for muscle differentiation and regeneration. BjsuV administration resulted in a significant reduction in all evaluated myogenic factors, demostrating a substantial decline of 70%, 30%, 34%, and 22% for MyF5, Pax7, MyoD, and myogenin, respectively, compared to the control group (Fig 2B–2E). In contrast, PBM irradiation demonstrated a notable reversal of this trend, eliciting a substantial increase in the expression of these myogenic factors by 74%, 49%, 38%, and 28%, respectively, when treated with PBM at 660 nm, and 104%, 73%, 52%, and 44%, respectively, when treated with PBM at 780 nm, compared to the venom-exposed group (Fig 2B–2E). Throughout the analysis, β-actin served as the loading control.

### Effect of PBM on cytokine levels in C2C12 cells

**Fig 3:**
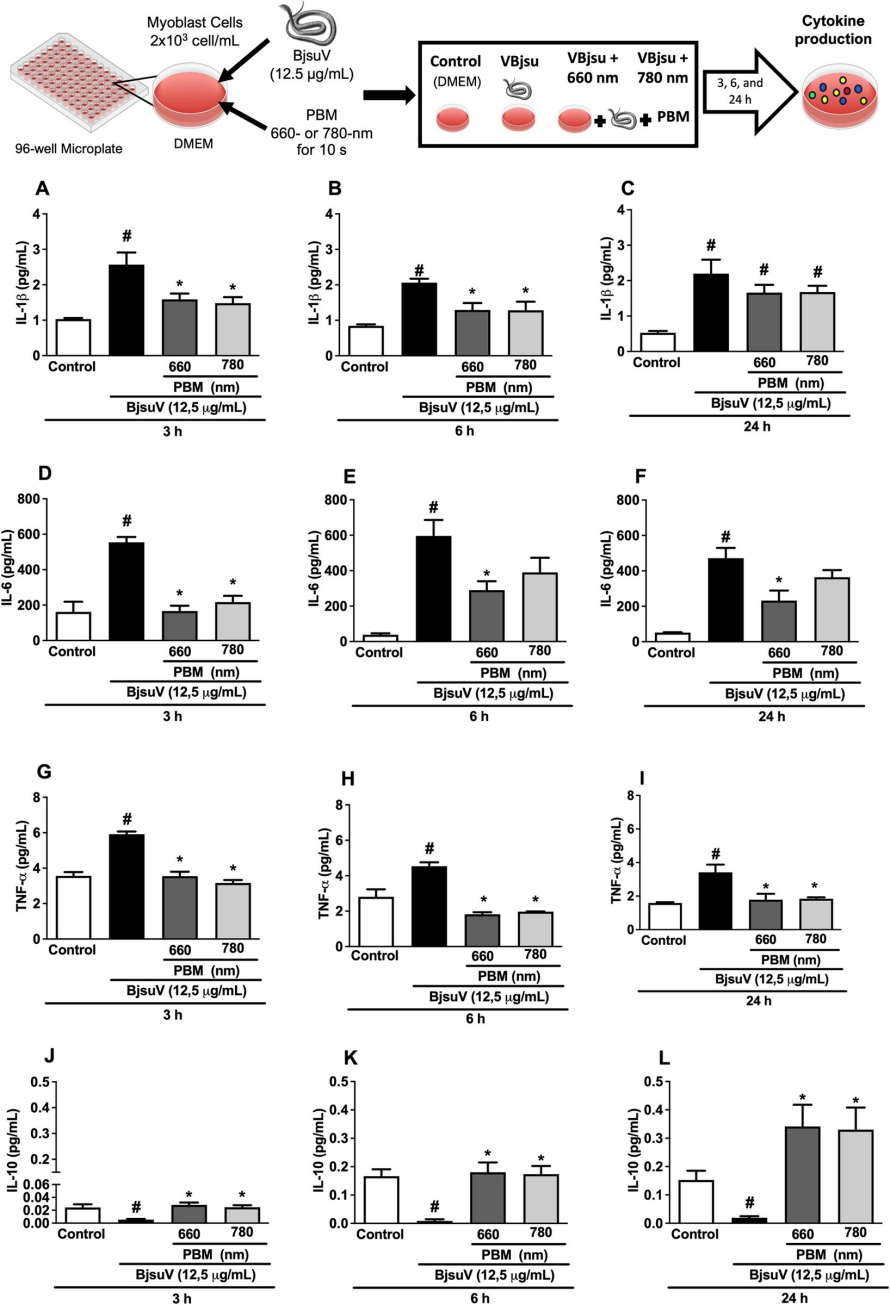
Effect of photobiomodulation on cytokine levels in C2C12 cells exposed to *B*. *jararacussu* venom. The supernatant of C2C12 cells was analyzed for interleukin-1β (IL-1β) (A, B, C), interleukin-6 (IL-6) (D, E, F), tumor necrosis factor-α (TNF-α) (G, H, I), and interleukin-10 (IL-10) (J, K, L) at 3, 6, and 24 hours. All the experiments were performed in triplicate and the data were expressed as mean ± SEM. #p < 0.05 vs. control group *p < 0.05 vs the venom-exposed group (ANOVA). BjsuV, *Bothrops jararacussu* venom; PBM, photobiomodulation. Figure diagram built using https://commons.wikimedia.org as source of the imagens or icons.

The concentrations of inflammatory cytokines (IL-1β, IL-6, TNF-α, and IL-10) in the supernatant of C2C12 cells were determined using ELISA (Fig 3). BjsuV induced a notable increase in all three proinflammatory mediators across all evaluated time points (3, 6, and 24 h) compared to the control group (all p values <0.001). PBM exhibited a reduction in IL-1β expression at the 6 hours (Fig 3A). PBM irradiation led to a decrease in IL-6 levels at 3 hours for both 660 nm and 780 nm wavelengths (Fig 3D). However, at 6 and 24 hours, only the 660 nm wavelength resulted in reduced IL-6 levels (Fig 3E and 3F). Significantly lower levels of TNF-α were observed in the PBM-treated groups compared to the BjsuV groups (Fig 3G–3I). Additionally, BjsuV significantly decreased the levels of the anti-inflammatory mediator IL-10 (Fig 3J–3L). Conversely, PBM treatment increased IL-10 levels consistently across all analyzed time periods and for both studied wavelengths (Fig 3J–3L).

### Effect of PBM on the expression of NF-κB and p38 MAPK

**Fig 4:**
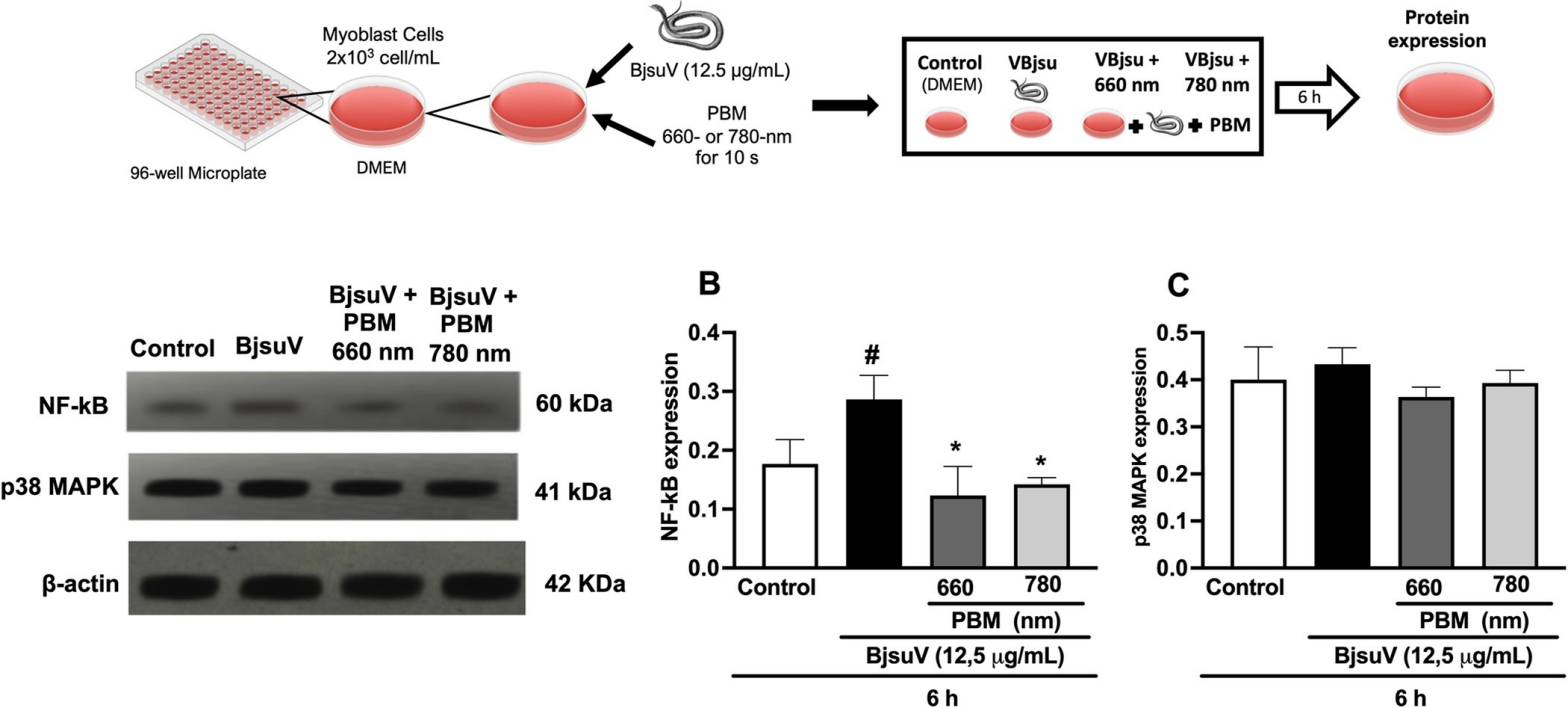
Effect of photobiomidulation on the expression of NF-kB and p38 MAPK. Representative immunoblot analyses and corresponding bar graphs depicting the protein levels of NF-kB (A and B), and p38MAPK (A and C) in C2C12 myoblast cells. The cells were incubated with 12.5 μg/mL of BjsuV, and the impact of photobiomodulation treatment was assessed after 6 hours. β-actin served as the loading control. All experiments were performed in triplicate, and the relative intensity of the bands was quantified. #p< 0.05 vs the control group; *p< 0.05 vs the venom-exposed group. BjsuV, *Bothrops jararacussu* venom; PBM, photobiomodulation. Figure diagram built using https://commons.wikimedia.org as source of the imagens or icons.

The levels of NF-κB and p38 MAPK in cell lysates were analyzed by western blotting. NF-κB expression was increased by BjsuV by 61%, compared with that of the control. PBM irradiation reduced the venom-induced NF-κB expression by 56% and 50% at 660 nm and 780 nm, respectively (Fig 4A and 4B). No difference in the expression of p38 MAPK was observed among the studied groups (Fig 4A and 4C).

## Discussion

Several studies have highlighted the significant impact of PBM therapy in mitigating skeletal muscle damage resulting from *Bothrops* envenomation (for a comprehensive review, see Silva et al. [4]). Despite these findings, the specific biological mechanisms underlying muscle repair attributed to PBM therapy in countering the local effects induced by Bothropic venom remain poorly understood. Therefore, our study aims to investigate some mechanisms associated with the effects of PBM therapy in myoblast cells in response to *B*. *jararacussu* venom.

After skeletal muscle injury, the process of muscle regeneration will develop, comprising successive phases, namely degeneration, regeneration, and reconstruction. Myoblasts are capable of migrating towards areas of muscle injury and regeneration [22] and the process of migration is a key step in the alignment of myoblasts for their subsequent fusion into multinucleated myotubes [23–25]. In our study we find that PBM irradiation with 660 nm and 780 nm promoted migration of murine C2C12 myoblasts in the presence of BjsuV. It is worth mentioning that within 24 hours, there were no viable cells in the venom-exposed group, confirming previous studies that showed the cytoprotective effect caused by PBM therapy [14,15,26]. In addition, our data suggest the potential enhancement of myoblast migration capacity, thereby contributing to the observed regenerative processes in muscle cells during *in vivo* assessments following venom exposure and PBM treatment [27–29].

Skeletal muscle regeneration occurs through a highly orchestrated process characterized by distinct phases, marked by the activation of diverse cellular and molecular responses that collectively contribute to the successful repair of muscle damage [13,30]. Following tissue injury, myogenesis is initiated, predominantly reliant on the expression of Pax genes, subsequently leading to the expression of key myogenic regulatory factors (MRFs), including MyoD, myogenin, and myogenic determination factor 5 (Myf5) [31]. To examine whether PBM irradiation influences myoblast cell activity in response to VBjsu, we performed western blotting for Pax7, a transcription factor expressed by both quiescent and proliferating satellite cells. Here, we demonstrated that PBM irradiation enhanced the protein level of Pax7 in response to venom-induced cytotoxicity when compared to venom treated cells, indicating activation of myoblast. Consistent to our results, a high expression on Pax-7 gene was observed in cell suspension from gastrocnemius muscle injected with Bothropstoxin I, a PLA2 toxin isolated from *B*. *jararacussu* venom, 4 hours after its injection [32]. Da Silva Neto Trajano et al. [33] demonstrated an increase in Pax7 gene expression in mouse C2C12 myoblast cultures 2 hours after exposure to PBM irradiation. The authors suggested that the PBM may be responsible for stimulating the proliferation of satellite cells. Additionally, the literature highlights that Pax-7 expression is an essential requirement for the proper functioning of satellite cells during regenerative myogenesis [34]. Therefore, the observed increase in Pax-7 in our study represents an important step in muscle regeneration induced by PBM following venom exposure.

Activated skeletal myoblasts exhibit the expression of key myogenic regulatory factors (MRFs), pivotal for reparative myogenesis [35]. In a previous study, we demonstrated that PBM protects C2C12 cells from the deleterious effects of *B*. *jararacussu* venom while concurrently fostering cell differentiation [15]. This was evidenced by an increase in the myogenic factors MyoD and myogenin, as assessed four days post-incubation with the venom [15]. The current study investigated into the immediate effects of PBM on the modulation of myogenic factors in C2C12 cells during the acute period. Our findings indicate that, within a 6-hour timeframe, the venom induces a significant reduction in all examined MRFs (Myf5, MyoD, and myogenin), while PBM irradiation in both in red and infrared wavelength significantly enhances the expression of these MRFs. The observed increase in these MRFs suggests enhanced myoblast differentiation and an accelerated process of muscle repair. In contrast to our study, Mesquita-Ferrari et al. [35], observed no elevation in MyoD mRNA expression in C2C12 cells exposed to laser irradiation (780 nm; 0.2 J). The observed difference in findings, may stem from differences in experimental methodology. In their study, Mesquita-Ferrari et al. [35] did not utilize a specific stimulus in the cells; rather, they solely observed whether photobiomodulation (PBM) induced an increase in cell differentiation. It is plausible that the absence of a specific cellular stimulus may have limited the action of PBM in stimulating cell differentiation pathways such as those involving MyoD and myogenin. In contrast, in our study, we incubated the cells with *B*. *jararacussu* venom, which induces necrosis in C2C12 cells [14]. This suggests that while PBM may have the potential to enhance cell differentiation, its effectiveness could be contingent upon the presence of an appropriate cellular stimulus to activate differentiation processes.

An inflammatory response is essential for the successful regeneration of skeletal muscle and correlates with the initial stages of myogenesis when satellite cells are first activated and begin to proliferate and differentiate [36]. Following muscle injury, an acute inflammatory reaction is initiated, a process that requires the infiltration of inflammatory cells, the release of growth factors, and pro- or anti-inflammatory cytokines [12]. Numerous studies have shown that Bothropic venoms induce the release of pro-inflammatory cytokines at the site of venom injection, thereby contributing to the exacerbation of local tissue damage [37–40]. In this study, we assessed the impact of PBM on the release of pro- and anti-inflammatory cytokines IL-6, IL-1β, TNF-α by C2C12 cells following incubation with venom. Our findings revealed an increase in IL-6, IL-1β, TNF-α levels in the group exposed to *B*. *jararacussu* venom. Extensive evidence supports the notion that numerous Bothropic venoms and the toxins derived from these venoms induce the release of cytokines [38–41]. In our study, PBM irradiation led to a significant reduction of the pro-inflammatory cytokines IL-16, IL-1β, TNF-α. Franco et al. [26] also demonstrated a reduction in IL-1β levels in endothelial cells irradiated with PBM and exposed to *B*. *jararaca* venom for up to 2 hours after the venom exposure, employing identical PBM parameters to those utilized in this study. In addition, Gouveia et al. [14] demonstrated a reduction of IL-1β and IL-6 in C2C12 cells following a 2 hours incubation with *B*. *jararaca*, *B*. *jararacussu* and *B*. *moojeni* venoms, and PBM treatment (660 nm, 4.4 J/cm^2^). In an *in vivo* model, concentrations of IL-6 and TNF-α were also diminished in the footpad of animals injected with *B*. *moojeni* venom and subsequently treated with PBM [41]. Given that bothrops venom can stimulate muscle cells to produce proinflammatory mediators, which could amplify tissue damage, and considering that PBM has the ability to decrease these productions, it is conceivable that, to some extent, PBM contributes to the muscle protection effect by mitigating the inflammatory response.

An important anti-inflammatory cytokine, IL-10, was also evaluated. IL-10 is recognized as a regulatory cytokine that plays a role in controlling the inflammatory process by inhibiting the secretion of proinflammatory cytokines [42]. PBM triggered an elevation in IL-10 levels. Initially, at 3 hours, IL-10 was observed at low levels, but its concentration progressively increased, up to 24 hours. Gouveia et al. [14] did not observe a significant change in IL-10 release following incubation with bothropic snake venom and PBM irradiation within 2 hours. The authors suggested that this early post-venom incubation period (2 h) and PBM treatment might be too early to detect changes in this cytokine. In alignment with our findings, Nadur-Andrade et al. [42] demonstrated a decrease in IL-10 mRNA levels in the footpad and spinal cord of mice injected with *B*. *moojeni* venom and irradiation with PBM significantly increased the levels of this cytokine 6 hours after venom injection. The increase in IL-10 highlights the positive effect of PBM treatment on the expression of this anti-inflammatory cytokine playing a significant role in the resolution of inflammation.

The MAPK family encompasses three principal members: extracellular signal-regulated kinases (ERK), p38, and c-Jun N-terminal kinase (JNK), representing distinct signaling cascades [43]. p38 MAPK, is typically activated by extracellular stress and proinflammatory cytokines, with a prominent role in the inflammatory process [44]. To examine a possible role of PBM between the reduced inflammation and MAPK pathway, we assessed p38 MAPK protein expression. No difference in p38 MAPK were observed among groups. Moreira et al. [45] showed that a myotoxin isolated from *B*. *asper* venom increases the expression of p38 MAPK and this effect is essential for the toxin induce the release of PGE2. Moreover, the literature demonstrated that PBM suppressed the phosphorylation of p38 MAPK from human bronchial epithelial cells stimulated with cigarette smoke exposure [46]. In addition, elevated expression of p38 MAPK has been associated with diabetic neuropathy, and PBM irradiation mitigate this condition [47]. We chose a 6-hour incubation period to assess p38 MAPK expression, guided by previous studies indicating a notable elevation in various cytokine levels induced by Bothropic venoms within this timeframe [14]. Thus, we selected this duration to investigate the potential activation of the p38 MAPK pathway in our experimental model, as we observed a significant increase in several cytokine levels, including IL-6, known to trigger p38 MAPK in muscle cells [48]. Although significant expression of this protein was not detected, it is plausible that expression may occur at other time intervals. This hypothesis will be examined in future studies.

The nuclear factor kB (NF-kB) constitutes a family of transcription factors pivotal in regulating diverse biological responses, particularly for its role in immune responses and inflammation [49]. In this context, our study speculates the hypothesis that the observed reduction in inflammation following PBM treatment in venom-exposed cells may be linked to the modulation of NF-kB. Consistent with other studies we observed an increase in the expression of NF-kB 6 hours after venom exposure [50–52]. Our findings demonstrated a significant suppressive effect of PBM on NF-kB, by both 660 nm and 7780 nm PBM irradiation. Notably, the impact of PBM on NF-kB in C2C12 cells following venom exposure aligns with the observed reduction in cytokine levels as assessed in this study. Indeed, as demonstrated in this study, the PBM irradiation effect on cytokine secretion may be due to the inhibition of NF-kB.

A limitation of the study is the absence of a control group with no stimulus (cells without venom) and irradiated with PBM. However, literature indicates that if cells are fully functional at the moment of irradiation or are growing in a serum-rich environment (usually 10% FBS), there is limited potential for PBM-mediated stimulation, and consequently, no therapeutic benefit will be observed [53]. Additionally, previous studies investigating the effects of PBM on C2C12 cells have consistently shown that PBM application within various dosimetric ranges (ranging from 3.8 J/cm2 to 17.5 J/cm2, using the same wavelength as in our study) does not significantly alter cell viability or proliferation [54,55]. Furthermore, drawing from our previous investigations conducted on endothelial cells, we have observed that PBM applied as a control did not impact cell viability, integrity, or cytotoxicity [26].

In conclusion, PBM therapy may serve as a novel therapeutic approach as an adjuvant treatment for the local effects of snakebite envenomation. This is based on its beneficial photophysical and photochemical effects, which counteract the observed cellular changes in these accidents. PBM therapy shows promise in increasing muscle regeneration and decreasing inflammatory process.
